# Self-Efficacy and Willingness to Implement Narrative Communication Interventions: Mixed Methods Study Among Breast Cancer Patients and Survivors at the University College Hospital, Ibadan, Nigeria

**DOI:** 10.1200/GO.22.39000

**Published:** 2022-05-05

**Authors:** Oluwaponmile Odukoya, Mojisola Oluwasanu, Ndidi Okunnuga, Omobolaji Ayandipo, Atara Ntekim, Oyedunni Arulogun, Olufunmilayo Olopade

**Affiliations:** ^1^ Department of Radiation Oncology, College of Medicine, University of Ibadan, Ibadan, Nigeria; ^2^ Department of Health Promotion and Education, University of Ibadan, Ibadan, Nigeria; ^3^ Department of Surgery, University of Medical Science Teaching Hospital, Akure, Nigeria; ^4^ Department of Surgery, College of Medicine, University of Ibadan, Ibadan, Nigeria; ^5^ Faculty of Public Health, College of Medicine, University of Ibadan, Ibadan, Nigeria; ^6^ Center for Global Health, University of Chicago, Chicago, IL

## Abstract

Globally, black women experience poorer breast cancer (BC) outcomes suggesting the need for effective health promotion approaches to increase perceived severity and improve screening practices for early detection. Although, narrative communication by patients or survivors are increasingly considered an effective approach for better BC outcomes, there is limited information on its use in Nigeria. This study assessed the self-efficacy and willingness of BC patients and survivors at the University College Hospital, Ibadan to implement narrative communication interventions.

**METHODS:**

This study utilized a mixed-method convergent parallel design. Five patients were recruited for in-depth interviews (IDI). A semi-structured questionnaire was used to collect data from 102 patients on perception and the generalized self-efficacy to implement narrative communication interventions. Data were analyzed using thematic analysis for qualitative and descriptive and inferential statistics for quantitative data.

**RESULTS:**

Mean age of the respondents was 49.3 ± 10.2 years. Over three quarters (79.4%) of respondents had good self-efficacy to implement narrative communication. Majority of respondents (87.3%) were willing to share lived experiences during narrative communication interventions. Over 90% (94.1%) agreed that health programs on BC anchored by patients and survivors will enable individuals make healthy decisions than interactions with clinicians only. Respondents' educational attainment was significantly associated with perception and self-efficacy to implement narrative communication intervention programs (*P* < .05). Patients opined that a pre-diagnosis discussion with a BC patient or survivor would have increased their perceived severity and susceptibility.

**CONCLUSION:**

This study highlights the potentials of BC patients and survivors as breast health educators sharing their lived experiences to empower and motivate women to act on health information on BC prevention and screening. Findings could guide the design of population-level interventions on BC prevention and screening.

